# Araloside C Prevents Hypoxia/Reoxygenation-Induced Endoplasmic Reticulum Stress via Increasing Heat Shock Protein 90 in H9c2 Cardiomyocytes

**DOI:** 10.3389/fphar.2018.00180

**Published:** 2018-04-17

**Authors:** Yuyang Du, Min Wang, Xuesong Liu, Jingyi Zhang, Xudong Xu, Huibo Xu, Guibo Sun, Xiaobo Sun

**Affiliations:** ^1^Institute of Medicinal Plant Development, Chinese Academy of Medical Sciences and Peking Union Medical College, Beijing, China; ^2^Beijing Key Laboratory of Innovative Drug Discovery of Traditional Chinese Medicine (Natural Medicine) and Translational Medicine, Beijing, China; ^3^Center of Research and Development on Life Sciences and Environmental Sciences, Harbin University of Commerce, Harbin, China; ^4^Academy of Chinese Medical Sciences of Jilin Province, Changchun, China

**Keywords:** Araloside C, hypoxia/reoxygenation injury, endoplasmic reticulum stress, heat shock protein 90, apoptosis

## Abstract

Araloside C (AsC) is a cardioprotective triterpenoid compound that is mainly isolated from *Aralia elata*. This study aims to determine the effects of AsC on hypoxia-reoxygenation (H/R)-induced apoptosis in H9c2 cardiomyocytes and its underlying mechanisms. Results demonstrated that pretreatment with AsC (12.5 μM) for 12 h significantly suppressed the H/R injury in H9c2 cardiomyocytes, including improving cell viability, attenuating the LDH leakage and preventing cardiomyocyte apoptosis. AsC also inhibited H/R-induced ER stress by reducing the activation of ER stress pathways (PERK/eIF2α and ATF6), and decreasing the expression of ER stress-related apoptotic proteins (CHOP and caspase-12). Moreover, AsC greatly improved the expression level of HSP90 compared with that in the H/R group. The use of HSP90 inhibitor 17-AAG and HSP90 siRNA blocked the above suppression effect of AsC on ER stress-related apoptosis caused by H/R. Taken together, AsC could reduce H/R-induced apoptosis possibly because it attenuates ER stress-dependent apoptotic pathways by increasing HSP90 expression.

## Introduction

According to the report released by the World Health Organization (2017), ischemic heart disease is the world’s biggest killers in 2015 worldwide, and it has remained the leading causes of death globally in the last 15 years. The most effective therapeutic intervention for reducing acute myocardial ischemic injury and limiting the size of myocardial infarction is timely and effective myocardial reperfusion using either thrombolytic therapy or primary percutaneous coronary intervention. However, myocardial reperfusion may itself contribute to further tissue damage, known as myocardial reperfusion injury (Hausenloy and Yellon, 2013; Binder et al., 2015). Molecular and cellular events underlying myocardial I/R injury are complex; nevertheless, scholars have provided increasing lines of evidence regarding the critical role of ER stress in pathological I/R injury process (Qi et al., 2007; Yu et al., 2016; Zhang et al., 2017).

Proper synthesis and correct folding of proteins in the ER are important for normal heart functions. During myocardial I/R, changes in cellular energy levels, redox state and Ca^2+^ concentration can disturb the ER protein-folding capacity and cause accumulation of unfolded proteins in the ER lumen; this condition, also called ER stress, activates UPR (Martindale et al., 2006; Gorman et al., 2012). The UPR is mediated through three transmembrane signal proteins: PERK, IRE1 and ATF6, which are inactivated by the ER chaperone GRP78. Once the unfolded proteins accumulating in the ER attract GRP78, the sensors start their signaling cascades (Bertolotti et al., 2000). The initial intent of the UPR is to adapt to the changing environment and re-establish normal ER function. However, under prolonged or excessive stress, the UPR can trigger pro-apoptotic signals, including CHOP, caspase-12 and JNK-dependent pathways (Gorman et al., 2012). Attenuation of ER stress induced apoptosis can protect the heart against I/R injury (Zheng et al., 2015; Gao et al., 2016; Yu et al., 2016), and inhibition of ER stress through certain proteins or signal pathways may represent a novel therapeutic mechanism. For example, modulating SERCA activity by activating of PI3K/Akt signaling may effectively suppress ER stress during I/R injury (Guo et al., 2013). Enhancing cardiac SIRT1 signaling by diallyl trisulfide can suppress PERK/eIF2α/ATF4/CHOP-mediated ER stress level, thereby reducing myocardial apoptosis and eventually preserving cardiac function (Yu et al., 2017). Upregulation of calpain-1 is sufficient to induce ER stress and apoptosis in cardiomyocytes, and inhibition of calpain-1 prevents ER stress and apoptotic cell death in H/R-stimulated cardiomyocytes (Zheng et al., 2015). Therefore, regulating ER stress to attenuate ER stress-induced apoptosis may become the focus of evolving strategies to ameliorate myocardial I/R injury.

HSP90, which accounts for approximately 1% of the total soluble proteins in resting mammalian cells (Latchman, 2001), is an ATP-dependent molecular chaperone responsible for managing protein folding and quality control in the crowded environment of the cell. HSP90 mediates cardioprotective effect by attenuating IRE1 activity in the ER through interacting with HAX-1 (Lam et al., 2013), implying its involvement in regulating UPR. Besides, researches also indicated the protection of HSP90 against myocardial I/R injury. Overexpression of HSP90 could attenuate cell apoptosis (Zhu et al., 2016) and reduce infarct size and myocardial dysfunction (Kupatt et al., 2004). Inhibition of HSP90 function or expression completely suppresses the protection of hypoxic preconditioning (Jiao et al., 2008). Therefore, modulation of HSP90 may be a plausible strategy for regulating the ER stress and alleviate the I/R injury.

*Aralia elata* (Miq.) Seem, a shrub belonging to the family *Araliaceae*, is mainly distributed in Northeast China, Far East Russia, Korea, and Japan (Shikov et al., 2016); this plant has been traditionally used in China as a tonic and medicine for the treatment of rheumatoid arthritis, diabetes and hepatitis (Zhang et al., 2013; Shikov et al., 2016). The main pharmacologically active ingredient in *A. elata* are saponins(Xu et al., 1997). Both *in vivo* and *in vitro* studies have shown that the total saponin extracted from *A. elata* can improve cardiac function (Wang et al., 2014b) and protect against myocardial ischemic (Sun et al., 2006), arrhythmia (Maslov and Guzarova, 2007; Maslov et al., 2009) and diabetic cardiomyopathy (Xi et al., 2009). AsC (**Figure 2A**) is one of the most abundant triterpenoid compounds isolated from the bark and root of *A. elata.* Our group previously reported that AsC can improve heart function following I/R injury possibly by binding to the HSP90 protein (Wang et al., 2017a). However, the further cardioprotective properties and whether and how HSP90 is involved in the protective effects of AsC are still unknown.

This study aims to determine: (1) whether AsC exerts protective effects on myocardial hypoxia-reperfusion (H/R) injury; (2) the effect of AsC on H/R-induced ER stress; and (3) the potential role of HSP90 and its modulation of ER stress in the cardioprotective effect of AsC.

## Materials and Methods

### Reagents and Materials

Araloside C was synthesized as previously reported (Wang et al., 2017a) at the Institute of Medicinal Plant Development (Beijing, China). Tunicamycin from Streptomyces was purchased from Sigma (St. Louis, MO, United States), and the 4-PBA (CAS.NO. 1821-12-1) was purchased from Sinopharm Chemical Reagent Co., Ltd (Beijing, China). All cell culture materials were from GIBCO (Grand Island, NY, United States). DMSO, MTT and 17-AAG were the products of Sigma Chemical Co. (St. Louis, MO, United States). The kits for determining LDH was obtained from Jiancheng Bioengineering Institute (Nanjing, China). The JC-1 was obtained from Beyotime Biotechnology Inc. (Beijing, China). The Alexa Fluor^®^ 488 annexinV/Dead Cell Apoptosis Kit, HSP90 siRNA and Lipofectamine^®^ RNAiMAX Reagent were acquired from Invitrogen (Carlsbad, CA, United States). The caspase-3 activity kit was purchased from BioVision, Inc. (Mountain View, CA, United States). RIPA lysis buffer, Protease Inhibitor Cocktail, BCA Protein Assay Kit, and Enhanced Chemiluminescence Western Blot Detection Kits were supplied by CWbiotech (Beijing, China). Primary antibodies against PARP, HSP90 and IRE1 were obtained from Abcam (Cambridge, United Kingdom). Primary antibodies against caspase-9, caspase-3, caspase-12, p-eIF2α and eIF2α were obtained from Cell Signaling Technology Inc. (Danvers, MA, United States). All other antibodies were purchased from Santa Cruz Biotechnology (Dallas, TX, United States). All chemical reagents were of at least analytical grade.

### Cell Culture and Hypoxia/Reoxygenation (H/R)

Rat embryonic cardiomyoblast-derived H9c2 cardiomyocytes were obtained from the Chinese Academy of Sciences Cell Bank (Shanghai, China) and cultured in high glucose DMEM supplemented with 10% (v/v) fetal bovine serum, 1% penicillin/streptomycin (v/v), and 2 mM L-glutamine. The cells were maintained in a humidified incubator with 95% air and 5% CO_2_ at 37°C. For all experiments, cells were plated at an appropriate density according to the experimental design and grown for 24 h to reach 70–80% confluence before experimentations began.

The H/R model was built using a modified process(Sun et al., 2013). Briefly, H9c2 cardiomyocytes were incubated at 37°C in an anaerobic glove box (Coy Laboratory, United States), where normal air was replaced by a combination of 5% CO_2_, 5% H_2_, and 90% N_2_, with the high glucose DMEM medium replaced by no-glucose DMEM to mimic ischemia. The cells were cultured under hypoxia for 6 h, and then removed to the regular incubator with the medium replaced by high glucose medium and were maintained for 12 h to mimic reperfusion.

### Protocols

Cultured H9c2 cardiomyocytes were randomly divided into different groups. In the control group, H9c2 cardiomyocytes were incubated in high glucose DMEM under normoxic conditions for equivalent durations. The H/R group was conducted as described in the preceding section. When employing TM to cause cell damage, H9c2 cardiomyocytes were incubated with TM (1 μg/ml) for 24 h. In the AsC- treated group (AsC+ H/R or AsC+ TM), H9c2 cardiomyocytes were treated with AsC (12.5 μM) for 12 h before H/R or TM. In 4-PBA treated group (PBA+ TM), the cells were incubated with 4-PBA (0.5 mM) for 12 h before exposed to TM. The inhibitor-treated groups (AAG+ AsC+ H/R) were processed similar to the AsC+ H/R group, but the cells were incubated with 0.1 μM 17-AAG for 1 h before treated with AsC.

The groups were clustered into three categories for experiment: the first category includes Control, H/R, H/R+ AsC and AsC groups; the second category includes Control, TM, AsC+ TM, and PBA+ TM groups; and the third category includes the Control, H/R, H/R+ AsC and AAG +AsC+ H/R groups.

### Cell Viability Analysis

Cell viability was determined by MTT assay. Briefly, H9c2 cardiomyocytes were plated on 96-well plates at a density of 1 × 10^4^ cells/well. After designated treatment, 20 μL of MTT (5 mg/mL) was added to each well and cells were incubated at 37°C for 4 h. Then, the culture medium with MTT was removed and the colored formazan crystals were dissolved in 150 μL of DMSO. Absorption was measured at 570 nm using a microplate reader (TECAN Infinite M1000, Austria). The viability of H9c2 cells in each well was presented as percentage of control cells.

### Measurement of LDH Release

H9c2 cardiomyocytes were cultured at 2 × 10^5^ cells/well in 6- well plates for 24 h. After H/R treatment with or without AsC pretreatment, the medium was collected to measure LDH release using a LDH assay kits.

### Measurement of Mitochondrial Membrane Potential (ΔΨm) Disruption

The change in mitochondrial membrane potential was detected by JC-1 staining. After treatment, the cells were incubated with JC-1 (2 μM final concentration) at 37°C in the dark for 30 min. Then the cells were washed three times with PBS and observed using fluorescence microscopy (EVOS^®^ FL Color, Life Technologies). For further analysis of the fluorescence intensity, the treated cells were harvested and incubated with JC-1 at 37°C in the dark for 30 min. The fluorescence intensity changes were analyzed using a microplate reader (TECAN Infinite M1000, Austria). The green JC-1 signal was detected at excitation wavelengths of 514 nm and emission wavelengths of 529 nm, the red signal was detected at excitation wavelengths of 585 nm and emission wavelengths of 590 nm.

### Flow Cytometric Detection of Apoptosis

The percentage of early Apoptosis rate was determined using the Annexin V/PI assay kit according to the manufacturer’s instruction. After treatment, the cells were harvested, washed with cold PBS, and incubated with 5 μL FITC-Annexin V and 1 μL PI working solution (100 μg/mL) in the dark for 15 min at room temperature. Cellular fluorescence was determined using a FACS Calibur flow cytometer (BD Biosciences, CA, United States). The early apoptosis rate was expressed as the ratio of Annexin V-positive/PI-negative cells to total cells.

### Analysis of Caspase-3 Activation

The activities of caspase-3 in H9c2 cardiomyocytes were measured using a fluorometric assay kit. Fluorescence intensity was detected using a microplate reader at excitation and emission wavelengths of 485 and 535 nm, respectively. The results are described as fold changes compared with the control group.

### siRNA Transfection

H9c2 cardiomyocytes were cultured in 6-well plates. When 50% confluence was reached, the cells were transfected with HSP90 siRNA (50 nM) or equivalent concentrations of control siRNA diluting by Lipofectamine RNAiMAX. The cells were transfected for 24h and incubated with 12.5 μM AsC for 12 h, followed with H/R treatment.

### Western Blot Analysis

Protein expression was analyzed by western blot as described previously (Sun et al., 2013). After treatment, H9c2 cardiomyocytes were harvested and lysed with cell lysis buffer containing 1% protease inhibitor cocktail. The lysate was centrifuged for 20 min at 12,000 *g* and 4°C to remove insoluble materials. Equal amounts of protein from each sample were separated by sodium dodecyl sulfate polyacrylamide gel electrophoresis (SDS-PAGE) and transferred onto a nitrocellulose membrane (Millipore Corporation, United States). After blocked with 5% (w/v) non-fat milk powder in Tris-buffer that contains 0.05% (v/v) Tween-20 (TBST) at room temperature for at least 2 h, the membranes were incubated overnight at 4°C with appropriate primary antibodies. Then the membrane was washed three times with TBST, incubated with secondary antibody at a 1: 2,000 dilution for 2 h, and washed with TBST again. Finally, the blots were visualized by enhanced chemiluminescence using a BioRad imaging system (Bio-Rad, Hercules, CA, United States). Densitometric analysis of the bands was performed using Gel Pro software (Media Cybernetics, Rockville, MD, United States).

### Statistical Analysis

The results were expressed as mean ± SD. Comparisons between groups were performed by Student’s *t*-test or one-way ANOVA followed by Newman-Keuls multiple comparison test using Prism 5.00 software (GraphPad Software, La Jolla, CA, United States). A *p*-value less than 0.5 was considered statistically significant. All experiments were repeated at least three times.

## Results

### Protective Effect of AsC on H/R-Induced Injury in H9c2 Cardiomyocytes

**FIGURE 1:**
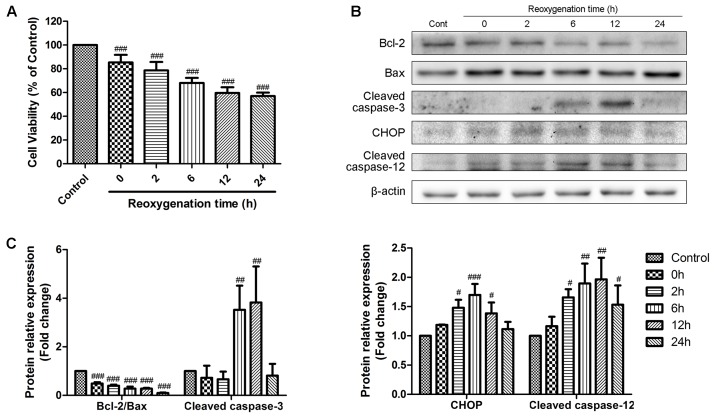
Effects of reoxygenation time on H9c2 cardiomyocytes cell viability and apoptotic proteins. H9c2 cardiomyocytes were subjected to 6h of hypoxia and then exposed to different duration (0, 2, 6, 12, 24 h) of reoxygenation. **(A)** Cell viability was measured by MTT assays. **(B,C)** The expression of cleaved caspase-3, Bcl-2, Bax, CHOP and cleaved caspase-12 were analyzed by western blotting, with representative bands quantified in the corresponding bar graph. β-actin expression was examined as the protein loading control. The values are expressed as the mean ± SD three independent experiments. ^#^*P* < 0.05 vs. Control, ^##^*P* < 0.01 vs. Control, ^###^*P* < 0.001 vs. Control.

We first examined the effects of reoxygenation time on cell viability and expression of representative apoptotic-related proteins. H9c2 cardiomyocytes were exposed to hypoxia for 6 h and reoxygenation for 0, 2, 6, 12, and 24 h. Cell viability was detected by MTT assay, and the percentage of viable cell in each group was calculated relative to control. As shown in **Figure 1A**, hypoxia for 6 h decreased the cell viability, and this effect was time-dependently promoted by reoxygenation, which was consistent with the literature report (Yu et al., 2016). We then conducted Western Blot analysis to evaluate the expression of proteins related to apoptosis at different reoxygenation times. The expression of cleaved caspase-3 increased after 6h of reoxygenation and decreased after 12 h; meanwhile, the ratio of Bcl-2 to Bax decreased since hypoxia (**Figures 1B,C**). The expression levels of CHOP and caspase-12, which are associated with ER stress-induced apoptosis signaling pathways, were rapidly induced after reoxygenation and reached the peak at 6 and 12 h, respectively (**Figures 1B,C**). These results indicated that ER stress was activated after reoxygenation, and apoptosis was initiated thereafter. After reoxygenation for 12 h, ER stress-induced apoptosis weakened. Therefore, hypoxia for 6 h and reoxygenation for 12 h were chosen as optimal conditions for subsequent experiments.

**FIGURE 2:**
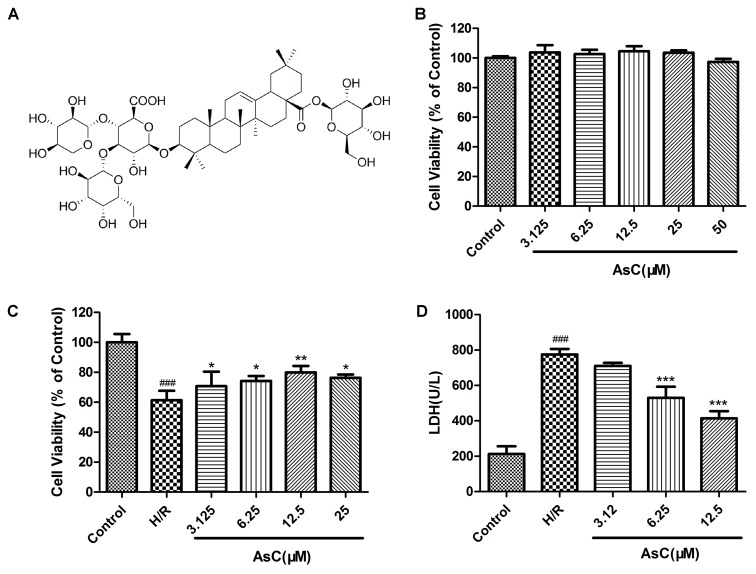
Effects of Araloside C on H/R-induced cell injury. **(A)** The chemical structure of AsC. **(B)** Cell viability of H9c2 cardiomyocytes incubated with different concentrations of AsC for 12 h. H9c2 cardiomyocytes were incubated with or without AsC for 12 h and then exposed to 6 h of hypoxia and 12 h of reoxygenation. **(C)** Cell viability was detected by MTT assay. **(D)** Effects of AsC on H/R-induced LDH leakage. The values are expressed as the mean ± SD three independent experiments. ^###^*P* < 0.001 vs. Control; ^∗^*P* < 0.05 vs. H/R group, ^∗∗^*P* < 0.01 vs. H/R group, ^∗∗∗^*P* < 0.001 vs. H/R group.

Next, we estimated the potential cardioprotective effects of AsC (**Figure 2A**) on H9c2 cardiomyocytes against H/R injury by using MTT and LDH assays. As shown in **Figure 2B**, AsC did not demonstrate any cytotoxicity after 12 h of pretreatment with virous concentrations. H9c2 cardiomyocytes exposed to H/R exhibited decreased cell viability compared to control, whereas pretreatment with different concentrations of AsC for 12 h conspicuously increased cell viability in a does-dependent manner (**Figure 2C**). The total saponins of Aralia elata (AS) (Sun et al., 2006; Wang et al., 2014b) and hydroxysafflor yellow A (HSYA) (Liu et al., 2012; Min and Wei, 2017), which were shown to be protective in myocardial ischemia/reperfusion injury, were used as positive controls here (**Supplementary Figure S3**). As a biomarker of cell death, LDH leakage was also detected. As shown in **Figure 2D**, H/R-induced LDH release was significantly decreased when the cells were pre-incubted with AsC (6.25- 12.5 μM) for 12 h.

### AsC Inhibits H/R-Induced Apoptosis in H9c2 Cardiomyocytes

**FIGURE 3:**
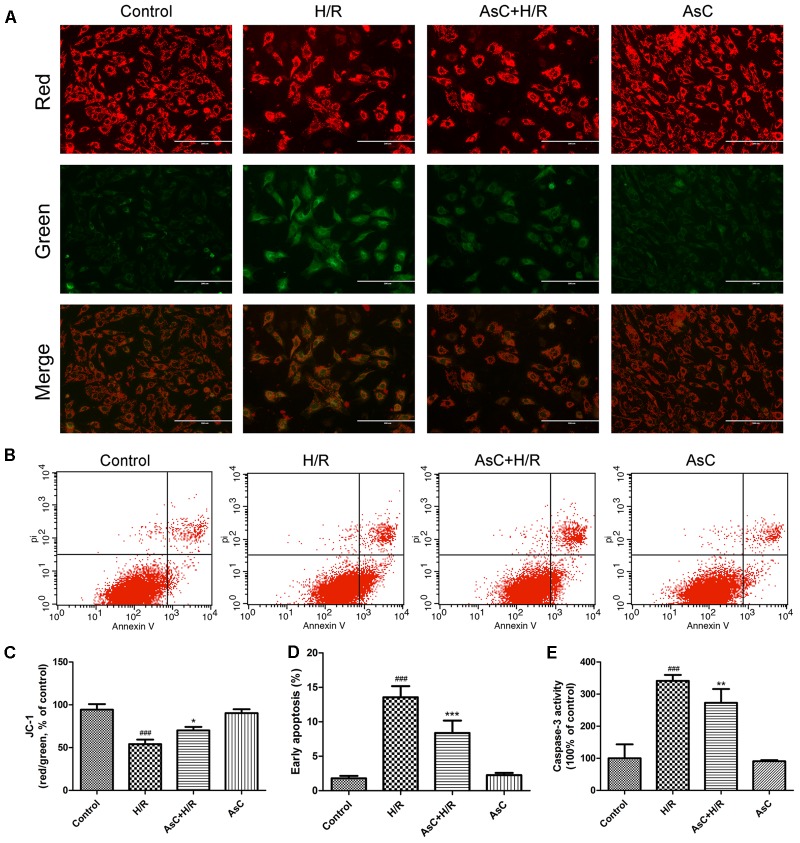
Araloside C protected against H/R-induced apoptosis in H9c2 cardiomyocytes. H9c2 cardiomyocytes were incubated with or without AsC for 12 h and then exposed to 6h of hypoxia and 12h of reoxygenation. **(A)** ΔΨm was assessed by fluorescence microscopy with JC-1 staining. **(B)** Apoptosis in H9c2 cardiomyocytes was analyzed by flow cytometry. **(C)** Quantitative analysis of mitochondrial membrane potential (ΔΨm) by plate reader. **(D)** Quantitative analysis of the percentages of early apoptotic cells. **(E)** caspase-3 activity was measured using a fluorometric assay. The values are expressed as the mean ± SD three independent experiments. ^###^*P* < 0.001 vs. Control; ^∗^*P* < 0.05 vs. H/R group, ^∗∗^*P* < 0.01 vs. H/R group, ^∗∗∗^*P* < 0.001 vs. H/R group.

Mitochondria plays a key role in I/R injury, and the disruption of mitochondrial membrane potential (ΔΨm) is an early event in the apoptotic cascade (Gustafsson and Gottlieb, 2008). Thus, we determined the effect of AsC on ΔΨm by JC-1 staining. The results showed that the H/R-treated group exhibited a significant decrease in the ratio of red/green fluorescence intensity, indicating ΔΨm dissipation; however, this effect was reversed by pretreatment with 12.5 μM AsC for 12 h (**Figures 3A,C**).

The Annexin V-FITC/PI staining assay demonstrated that the number of early apoptotic cells significantly increased in H/R-treated H9c2 cardiomyocytes compared with that in the control group, while incubation with 12.5 μM AsC effectively alleviated H/R-induced early apoptosis (**Figures 3B,D**).

**FIGURE 4:**
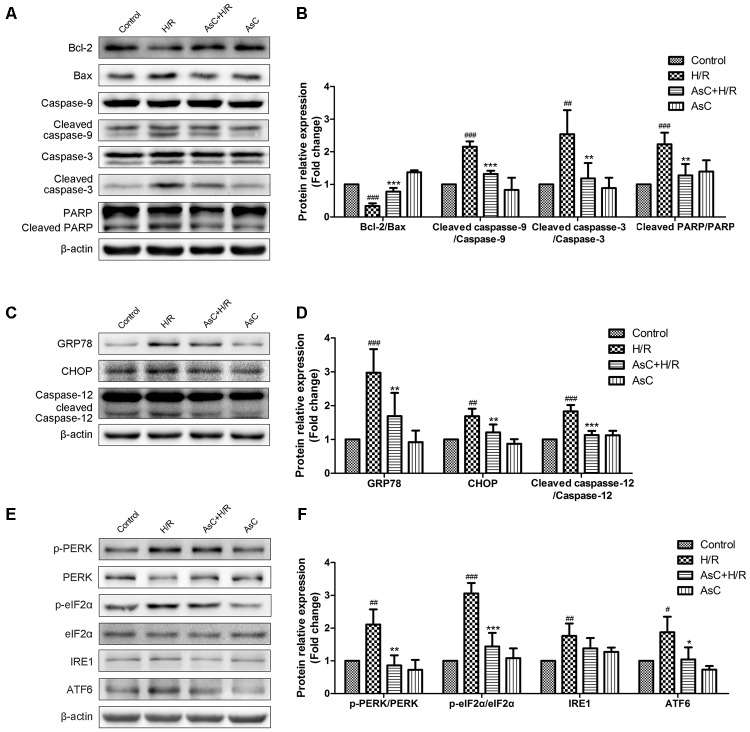
Araloside C suppressed H/R-induced apoptosis and ER stress in H9c2 cardiomyocytes. H9c2 cardiomyocytes were incubated with or without AsC for 12 h and then exposed to 6 h of hypoxia and 12 h of reoxygenation. **(A,B)** The expression of Bcl-2, Bax, caspase-9, caspase-3 and PARP were analyzed by western blotting, with representative bands quantified in the corresponding bar graph. **(C,D)** The ER stress marker GRP78 and ER stress-related apoptotic proteins (CHOP and caspase-12) were evaluated by western blot analysis. **(E,F)** The expression level of UPR pathway proteins were detected using western blot analysis and the relative protein expression of p-PERK to PERK, p- eIF2α to eIF2α, IRE1 and ATF6 to β-actin are expressed in the bar graphs. The values are expressed as the mean ± SD three independent experiments. ^#^*P* < 0.05 vs. Control. ^##^*P* < 0.01 vs. Control. ^###^*P* < 0.001 vs. Control; ^∗^*P* < 0.05 vs. H/R group, ^∗∗^*P* < 0.01 vs. H/R group, ^∗∗∗^*P* < 0.001 vs. H/R group.

To further confirm the anti-apoptotic effects of AsC, we evaluated the expression of apoptosis-related proteins by western blot. As shown in **Figures 4A,B**, H/R decreased the ratio of Bcl-2/Bax, increased the expression level of cleaved caspase-9, cleaved caspase-3 and PARP. These changes were reversed by AsC treatment. We also assessed caspase-3 activity by fluorimetric assay. Consistent with the effect on the expression of cleaved caspase-3, AsC pretreatment for 12h remarkably reduced caspase-3 activation induced by H/R (**Figure 3E**).

All these results indicated that AsC protected the cardiomyocytes against H/R injury partly by attenuating apoptosis activation.

### AsC Suppressed H/R-Induced UPR Pathways and ER Stress-Related Apoptosis in H9c2 Cardiomyocytes

The ER stress-responsive marker GRP78 and ER stress-dependent apoptotic proteins, namely CHOP and caspasse-12, were evaluated through Western Blot analysis to determine the effect of AsC on H/R-induced ER stress. As shown in **Figures 4C,D**, pharmacological intervention with 12.5 μM AsC for 12 h significantly suppressed the H/R-induced upregulation of GRP78 and the expression of the pro-apoptotic proteins (CHOP and caspase-12) compared with those in the control.

**FIGURE 5:**
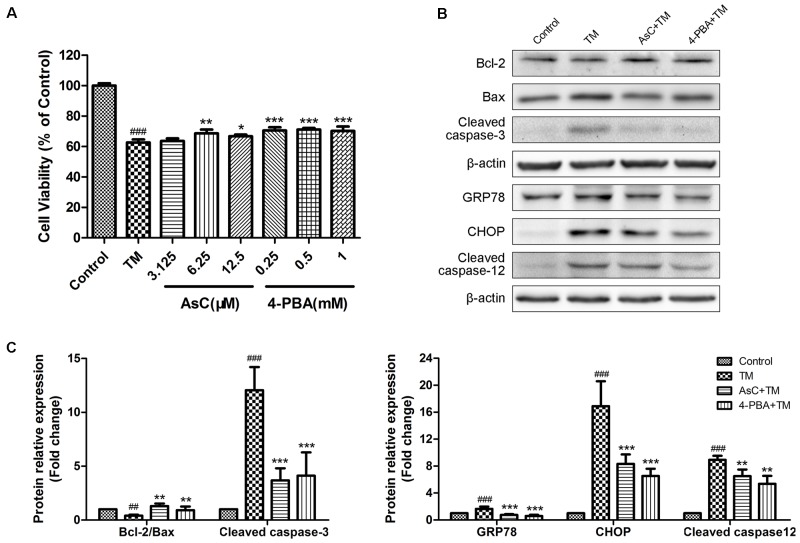
Araloside C inhibited TM-induced cell death and ER stress in H9c2 cardiomyocytes. **(A)** H9c2 cardiomyocytes were incubated with different concentrations of AsC (3.125, 6.25 and 12.5 μM) or 4-PBA (0.25, 0.5, and 1 mM) for 12 h, followed by treatment with TM (1 μg/ml) for another 24 h. Cell viability was detected by MTT assay. **(B,C)** H9c2 cardiomyocytes pretreated with AsC (12.5 μM) or 4-PBA (1 mM) for 12 h were exposed to TM (1 μg/ml) for 24 h. Representative apoptotic proteins (cleaved caspase-3, Bcl-2 and Bax), as well as ER stress marker GRP78 and ER stress-related apoptotic proteins (CHOP and cleaved caspase-12) were evaluated by western blot analysis. The values are expressed as the mean ± SD three independent experiments. ^##^*P* < 0.01 vs. Control. ^###^*P* < 0.001 vs. Control; ^∗^*P* < 0.05 vs. H/R group, ^∗∗^*P* < 0.01 vs. H/R group, ^∗∗∗^*P* < 0.001 vs. H/R group.

To further confirm whether or not AsC protected cardiomyocytes by modulating ER stress-dependent apoptosis, we used a typical ER stress inducer tunicamycin (TM) to stimulate ER stress and induce cell damage. We assessed the effect of AsC on TM-induced ER stress by MTT assay and Western Blot analysis with 4-PBA as positive control. As shown in **Figure 5**, treatment of 1 μg/ml TM for 24 h decreased the cell viability and the ratio of Bcl-2/Bax and promoted the activation of caspase-3, CHOP, caspase-12 and GRP78. Moreover, both AsC and 4-PBA preconditioning greatly reduced TM-induced injury and ER stress-related apoptosis. These results suggested that ER stress participated in H/R-induced injury, and the cardioprotective effect of AsC may partly rely on its suppression on ER stress-related apoptosis.

To investigate the mechanism whereby AsC prevents cell death in response to H/R-induced ER stress, we next evaluated the effects of AsC on the UPR signal in stressed cardiomyocytes. Western blot analysis showed that hypoxia for 6 h and reoxygenation for 12 h activated the three arms of the UPR compared to control. In comparison with H/R treated cells, 12 h pretreatment with 12.5 μM AsC remarkably reduced the phosphorylation of PERK and eIF2α and decreased ATF6 expression, while there was no significant effect on the increased expression of IRE1 (**Figures 4E,F**).

### Inhibition of HSP90 Attenuated the Cardioprotective Effects of AsC

**FIGURE 6:**
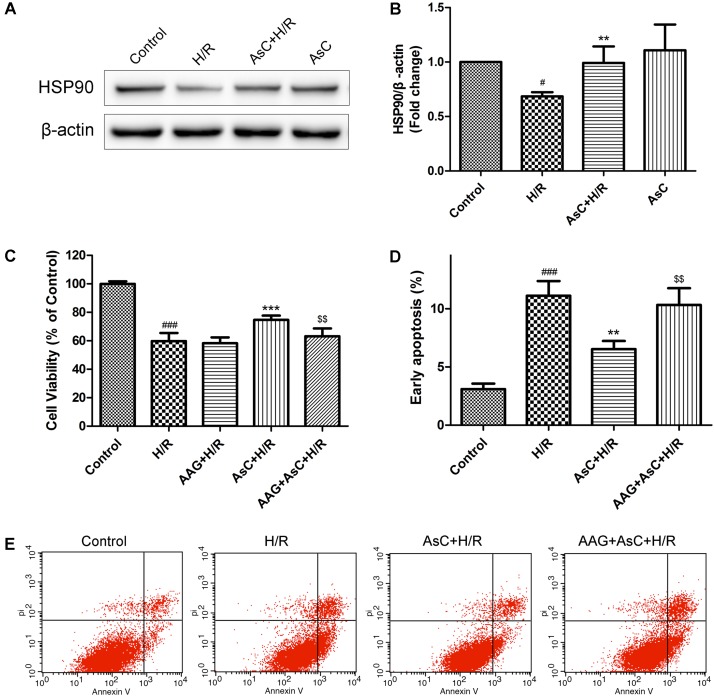
Araloside C protected H9c2 cardiomyocytes against H/R-induced cell death by increasing the expression of HSP90. **(A,B)** H9c2 cardiomyocytes were incubated with or without AsC for 12 h and then exposed to 6h of hypoxia and 12 h of reoxygenation. The expression of HSP90 were analyzed by western blotting. Then 17-AAG was used to inhibited HSP90 in order to explore the involvement of HSP90 in the cardioprotective effect of AsC. H9c2 cardiomyocytes were incubated with 17-AAG (0.1 μM) for 1 h before treated with AsC. **(C)** Cell viability was detected by MTT assay. **(D)** Quantitative analysis of the percentages of early apoptotic cells. **(E)** Apoptosis in H9c2 cardiomyocytes was analyzed by flow cytometry. The values are expressed as the mean ± SD three independent experiments. ^#^*P* < 0.05 vs. Control, ^###^*P* < 0.001 vs. Control; ^∗∗^*P* < 0.01 vs. H/R group, ^∗∗∗^*P* < 0.001 vs. H/R group; ^$$^*P* < 0.01 vs. AsC+ H/R.

We first detected the effects of AsC on HSP90 expression levels in H/R-treated H9c2 cardiomyocytes. Consistent with our previous reports (Wang et al., 2017a), AsC treatment significantly inhibited the H/R-induced down-regulation of HSP90 expression (**Figures 6A,B**).

**FIGURE 7:**
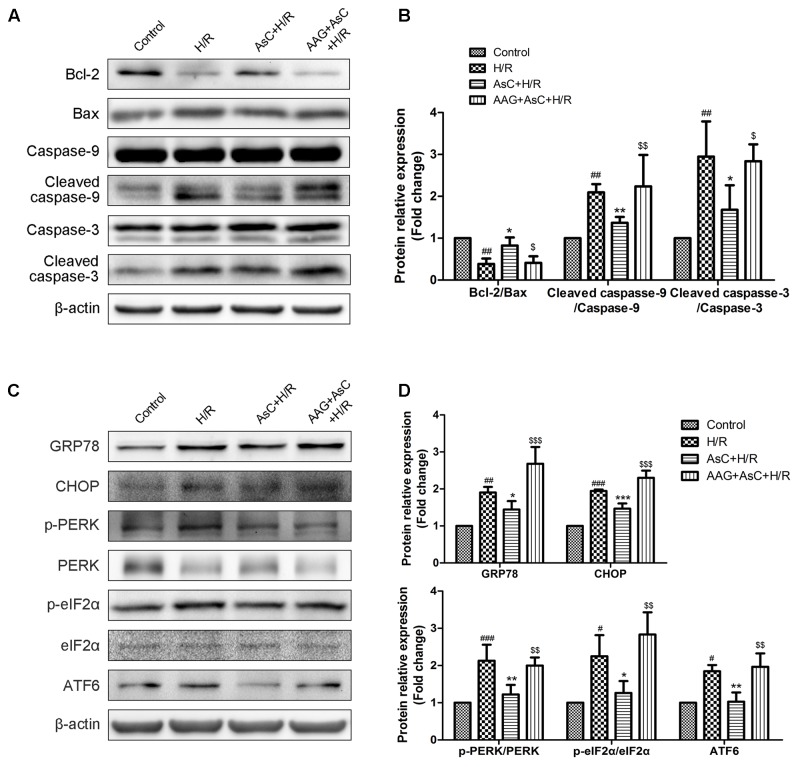
Inhibition of HSP90 by 17-AAG reversed the suppressive effect of AsC on H/R-induced ER stress. H9c2 cardiomyocytes were incubated with 17-AAG (0.1 μM) for 1 h and treated with AsC (12.5 μM) for another 12 h, followed by 6 h of hypoxia and 12 h of reoxygenation. **(A,B)** Expression of Bcl-2, Bax, caspase-9 and caspase-3 were analyzed by western blotting, with representative bands quantified in the corresponding bar graph. **(C,D)** ER stress-associated proteins (GRP78, CHOP, p-PERK/PERK, p-eIF2α/eIF2α and ATF6) were detected using western blot analysis, with representative bands quantified in the corresponding bar graph. The values are expressed as the mean ± SD three independent experiments. ^#^*P* < 0.05 vs. Control, ^##^*P* < 0.01 vs. Control, ^###^*P* < 0.001 vs. Control; ^∗^*P* < 0.05 vs. H/R group, ^∗∗^*P* < 0.01 vs. H/R group, ^∗∗∗^*P* < 0.001 vs. H/R group; ^$^*P* < 0.05 vs. AsC+ H/R, ^$$^*P* < 0.01 vs. AsC+ H/R, ^$$$^*P* < 0.001 vs. AsC+ H/R.

Then we utilized 17-AAG, a selective inhibitor of HSP90, to further investigate the contribution of the elevated expression of HSP90 to AsC-mediated protection against H/R-induced ER stress and apoptosis. Compared with that in the H/R group, pretreatment with AsC increased the cell viability and the ratio of Bcl-2/Bax, but significantly reduced the rate of early apoptosis and the expression level of cleaved caspase-9 and caspase-3. However, these effects were reversed by pretreatment with 0.1 μM 17-AAG for 1 h (**Figures 6C–E**, **7A,B**).

**FIGURE 8:**
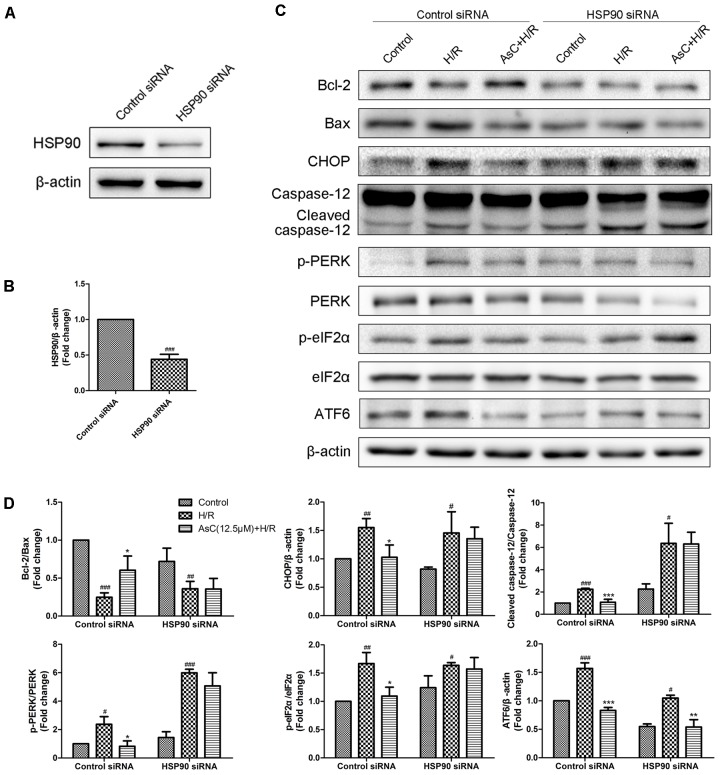
Interference of HSP90 by siRNA reversed the suppressive effect of AsC on H/R-induced ER stress. **(A,B)** H9c2 cardiomyocytes were transfected with control siRNA or HSP90 siRNA for 24h. These cells were then harvested, lysed, and analyzed for HSP90 protein expression levels by western blotting. **(C,D)** H9c2 cardiomyocytes transfected with control siRNA or HSP90 siRNA were incubated with AsC (12.5 μM) for 12 h and then subjected to 6h of hypoxia and 12 h of reoxygenation. The expression levels of ER stress-assoceated proteins were detected using western blot analysis. The relative protein expression of Bcl-2 to Bax, CHOP, caspase-12, ATF6 to β-actin, p-PERK to PERK and p- eIF2α to eIF2α were expressed in the bar graphs. The values are expressed as the mean ± SD three independent experiments. ^#^*P* < 0.05 vs. Control, ^##^*P* < 0.01 vs. Control, ^###^*P* < 0.001 vs. Control; ^∗^*P* < 0.05 vs. H/R group, ^∗∗^*P* < 0.01 vs. H/R group, ^∗∗∗^*P* < 0.001 vs. H/R group.

Additionally, the inhibitive effect of AsC on H/R-induced ER stress was abolished when the H9c2 cardiomyocytes were pretreated with 17-AAG, which were demonstrated by the upregulated protein expression level of p-PERK to PERK, p- eIF2α to eIF2α, CHOP and ATF6 to β-actin in 17-AAG pretreated group compared with those in AsC + H/R group (**Figures 7C,D**). Consistent with the effect of 17-AAG, interfering the expression of HSP90 with siRNA also reversed the suppressive effect of AsC on H/R-induced ER stress apoptosis. Interestingly, transfected with HSP90 siRNA in H9c2 cardiomyocytes removed the inhibition of AsC on the phosphorylation of PERK and eIF2α, but had no influence on the suppressive effect on ATF6 (**Figure 8**). Therefore, AsC inhibited H/R-induced over-activation of ER stress by increasing the expression level of HSP90.

## Discussion

Ischemic myocardial injury occurs under many clinical conditions, such as heart transplantation, cardiac bypass, and coronary stenting after acute myocardial infarction. However, the concept of the so-called “reperfusion injury” has not been totally accepted until recent years (Zhao et al., 2003; Bopassa, 2012). In the present study, we demonstrated that reperfusion for 2–24 h significantly decreased the cell viability and the expression ratio of Bcl-2/Bax compared with reperfusion for 0 h group, namely, ischemic group; reperfusion for 6–12 h promoted the activation of caspase-3 (**Figure 1**). These findings are consistent with previous studies (Liu et al., 2015) and confirmed the existence of reperfusion injury. Both necrosis and apoptosis were shown to be involved in H/R-induced cell death (**Supplementary Figure S1**) (McCully et al., 2004; Koshinuma et al., 2014). However, as shown in **Supplementary Figure S2**, cells in Annexin V positive and PI negative (AV+/PI-, represented early apoptosis) were markedly higher than that in Annexin V positive and PI positive (AV+/PI+, represented late apoptosis and necrosis) after H/R treatment, suggesting that apoptosis plays a critical role in pathogenesis of I/R injury (Eefting et al., 2004; Liu et al., 2015).

An increasing number of studies have suggested that ER stress plays a crucial role in I/R-induced apoptosis of cardiomyocytes (Lam et al., 2013; Zhang et al., 2017). ER stress sensors initiating the UPR are IRE1, ATF6, and PERK which can inhibit general protein translation through the phosphorylation of eIF2α (Harding et al., 2000). Although the UPR is a defense mechanism directed toward cellular adaptation to alleviate the unfolded protein load, prolonged stress is associated with the activation of apoptotic proteins like CHOP and caspase-12 (Gorman et al., 2012). In the present study, the protein level of the ER stress marker GRP78 was significantly increased and the activation of ATF6, IREα, PERK and eIF2α as well as the downstream apoptosis factors CHOP and caspase-12 were promoted in H9c2 cardiomyocytes subjected to H/R, indicating that ER stress was excessively activated and partly contributed to the cell apoptosis during H/R. However, this H/R-induced processing was remarkably suppressed by AsC pretreatment. Interestingly, AsC had no influence on H/R-induced upregulation of IRE1 (**Figures 4E,F**), implying that AsC might selectively inhibit the PERK and ATF6 branch in the UPR. Some research found that cell death under ER stress depended on the core mitochondrial apoptosis pathway, which is regulated by the Bcl-2 protein family (Tait and Green, 2010). CHOP, which is positively controlled by the PERK- eIF2α-ATF4 axis, promotes both the transcription of BIM and the downregulation of Bcl-2 expression, contributing to the induction of apoptosis (Li et al., 2014). Consistent with previous reports, our study confirmed that the reversal of ER stress by AsC was accompanied by increased Bcl-2/Bax ratio and reduced apoptotic cardiomyocytes (**Figures 4A,B**).

To further identify the effect of AsC on ER stress, we treated H9c2 cardiomyocytes with tunicamycin (TM), a well characterized ER stress inducing agent that can initiate ER stress-induced apoptosis by preventing glycosylation and protein folding and by inducing prolonged ER stress in treated cells (Wang et al., 2017b). 4-PBA is a chemical chaperone that improves ER folding capacity and facilitating the trafficking of mutant proteins (Jian et al., 2016a). It shows protective effects against ER stress-induced neuronal and myocardial cell death (Mimori et al., 2012; Jian et al., 2016a,b). Hence, 4-PBA was employed here as positive control. Both AsC and 4-PBA significantly ameliorated H/R-induced cell apoptosis and ER stress (**Figure 5**), which manifested the direct inhibitory effect of AsC on ER stress.

The total saponins are suggested to be the main pharmacological active ingredients of *A. elata* and exhibit anti-myocardial ischemic, anti-apoptotic and anti-inflammatory properties (Sun et al., 2006; Wang et al., 2014b; Chen et al., 2015). They also the main pharmacodynamic ingredient of Long Ya Guan Xin Kang Jiao Nang (Chinese name), a Chinese pharmaceutical drug developed for the treatment of angina pectoris (Chen et al., 2015). Our laboratory previously found that the total saponins extracted from *A. elata* and a saponin monomer can inhibit ER stress-related apoptosis and play protective roles in non-alcoholic steatohepatitis (Luo et al., 2015) and I/R injury (Wang et al., 2014a, 2015), respectively. As one of the major triterpenoid compounds isolated from *A. elata*, AsC can attenuate H/R induced ER stress-dependent apoptosis. Therefore, these results not only partially suggest the underlying mechanism of the cytoprotective effect of AsC, but also provide theoretical supports for the clinical application and further development of *A. elata*.

HSP90, a well-characterized, conserved and essential chaperone protein in eukaryotes, is shown to interact with the cytoplasmic parts of PERK (Marcu et al., 2002). Agents that has higher affinity, such as HSP90 inhibitors, may destabilize the activity of PERK by displacing them from HSP90 and subsequently regulate the ER stress (Marcu et al., 2002; Lam et al., 2013). A growing body of evidence implicates that HSP90 plays a critical role in the protective effect of myocardial ischemic preconditioning (Jiao et al., 2008; Amour et al., 2009) and against I/R injury (Kupatt et al., 2004; Zhu et al., 2016). Our oratory previously revealed that AsC was able to bind to the HSP90 protein *in vitro* in a dose-dependent manner with the KD 29 μM (Wang et al., 2017a).The current results confirmed that AsC significantly attenuated the apoptosis and the down-regulation of HSP90 expression induced by H/R H9c2 cardiomyocytes. By contrast, inhibition of HSP90 by 17-AAG, a well-known inhibitor of HSP90, notably blocked the AsC-induced cardioprotective effect against H/R injury (**Figures 6C–E**). In present study, we also found that 17-AAG and interference of HSP90 by siRNA remarkably reversed the suppression effect of AsC on H/R-induced UPR and ER stress-dependent apoptosis (**Figures 7**, **8**), indicating that the myocardial protective effect of AsC was likely due to its ability to increase HSP90. It was notable that HSP90 siRNA abolished the inhibition effect of AsC on PERK/eIF2α pathway, while it did not affect that on ATF6. Therefore, AsC probably protected against H/R injury through mediating the interaction between HSP90 and PERK. However, further research is needed to investigate and confirm the interaction of AsC with ER stress signal pathways and HSP90.

The following study limitations should be acknowledged. In this study, we showed that AsC can alleviate H/R-induced ER stress-associated cardiomyocyte apoptosis as it can downregulate the expression of CHOP and inhibit caspase-12 cleavage. Caspase-12 is shown to be a ER resident caspase and activated by ER stress. Mice deficient in caspase-12 are resistant to ER-induced apoptosis (Nakagawa et al., 2000). Terai K and collaborators have demonstrated that knockdown of caspase-12 through small interfering RNA (siRNA) resulted in decreased expression of cleaved PARP following exposure to hypoxia (Terai et al., 2005). By contrast to these studies, other work using caspase-12-/- MEFs observed no resistance to ER stressors such as thapsigargin (Saleh et al., 2006). These results suggest that caspase-12 may not be a predominant apoptotic pathway activated by ER stress in some types of cells and the predominant apoptotic pathway may differ among cell type and differentiation stage (Momoi, 2004). Moreover, it has been reported that caspase-12 is phylogenetically one of the inflammatory group caspases (Martinon and Tschopp, 2004). It is generally recognized as a negative regulator of the inflammatory response because it inhibits the activation of caspase-1 in inflammasome complexes, thereby modulating the production of IL-1b and IL-18 (Hermel and Klapstein, 2011). Murine caspase-12 deficiency confers resistance to sepsis and its presence exerts a direct suppressive effect on caspase-1, resulting in enhanced vulnerability to bacterial infection and septic shock (Saleh et al., 2006). In our research, we didn’t use caspase-12 inhibitor to further focus on the role of caspase-12 in I/R mediated apoptosis, so we cannot rule out the possibility that caspase-12 might be involved in inflammatory response in our experiment and the protective effect of AsC against H/R injury might be partially resulted from its inhibition of inflammatory. Further investigations need to be conducted to determine the role of caspase-12, as well as AsC, on H/R-induced inflammatory.

**FIGURE 9:**
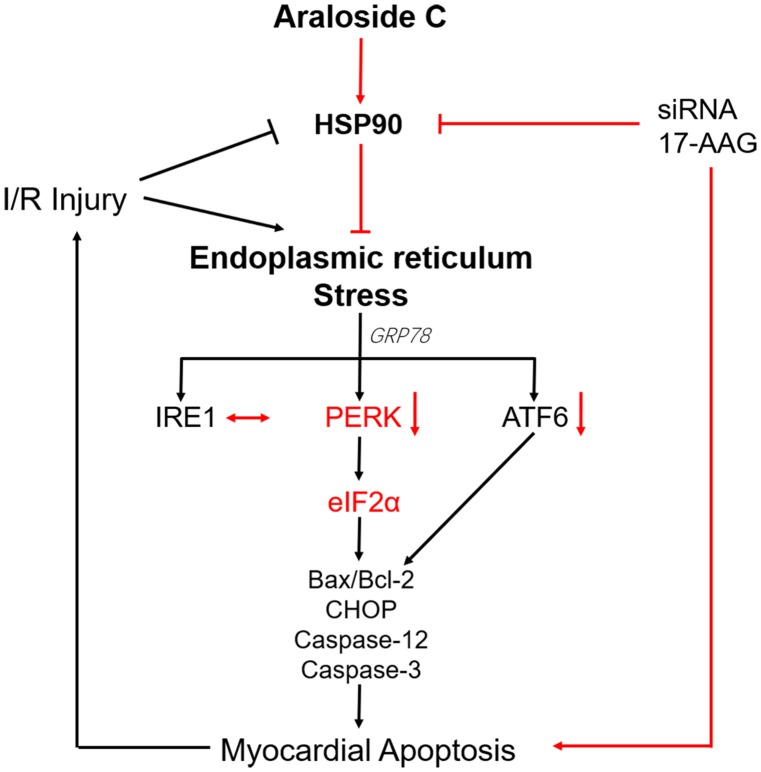
Schematic representation of AsC-mediated cardioprotective effect against H/R injury. AsC inhibited ER stress-dependent apoptotic pathways though attenuating PERK/eIF2α signal pathway in ER stress. This effect was mediated by the increase of HSP90 expression. 17-AAG and siRNA abolished the protective effect of AsC against H/R injury.

In summary, our data demonstrated that AsC protected against H9c2 cardiomyocytes against H/R injury, at least in part, by alleviating ER stress-associated apoptosis via increasing the expression of the HSP90 protein (**Figure 9**). It might provide some references for the development of AsC into cardiovascular drugs.

## Author Contributions

XS, GS, and HX supervised the project. YD, MW, XL, and JZ designed the detailed experiments, performed the study, and collected and analyzed data. HX and XX contributed reagents and materials. YD and MW wrote or contributed to the writing and revision of the manuscript. All authors commented the study and approved the final manuscript.

## Conflict of Interest Statement

The authors declare that the research was conducted in the absence of any commercial or financial relationships that could be construed as a potential conflict of interest.

## Supplementary Material

FIGURE S1 Effects of zVAD-fmk (zVAD, apoptosis inhibitor) and Necrostatin-1 (Nec-1, necrosis inhibitor) on H/R-induced cell injury. H9c2 cardiomyocytes were treated with different concentrations of zVAD and Nec-1 for 12 h, followed by 6 h of hypoxia and 12 h of reoxygenation. Cell viability was measured by MTT assays. The values are expressed as the mean ± SD four independent experiments. ^###^*P* < 0.001 vs. Control; ^∗∗∗^*P* < 0.001 vs. H/R group.

FIGURE S2 Quantitative analysis of the percentages of Annexin V positive and PI negative (AV+/PI-) cells and Annexin V positive and PI positive (AV+/PI+) cells with or without H/R treatment. Annexin V/PI assay kit was used and cellular fluorescence was determined by flow cytometer. The values are expressed as the mean ± SD four independent experiments. ^###^*P* < 0.001 vs. Control; ^∗∗∗^*P* < 0.001 vs. H/R group in AV+/PI-) cells.

FIGURE S3 Effects of AsC on H/R-induced cell injury. H9c2 cardiomyocytes were treated with different concentrations of AsC and Nec-1 for 12 h, followed by 6 h of hypoxia and 12 h of reoxygenation. Total saponins of *Aralia elata* (AS, 12.5 μg/ml) and hydroxysafflor yellow A (HSYA, 3 μM) were used as positive controls. Cell viability was measured by MTT assays. The values are expressed as the mean ± SD four independent experiments. ^###^*P* < 0.001 vs. Control; ^∗∗^*P* < 0.01 vs. H/R group, ^∗∗∗^*P* < 0.001 vs. H/R group.

## Abbreviations

17-AAG: 17-allylamino-17-demethoxygeldanamycin
4-PBA: 4-phenylbutyric acid
AsC: Araloside C
ATF6: activating transcription factor 6
CHOP: CCAAT/enhancer binding protein homologous protein
DMSO: Dimethylsulfoxide
eIF2α: eukaryotic translation initiator factor 2α
ER: endoplasmic reticulum
GRP: Glucose Regulated Protein
H/R: hypoxia-reoxygenation
HSP: heat shock protein
I/R: ischemic/reperfusion
IRE1: inositol-requiring enzyme 1
LDH: lactate dehydrogenase
MTT: 3-(4,5-dimethylthiazol-2-yl)-2,5-diphenyltetrazolium bromide
PARP: poly ADP-ribose polymerase
PERK: pancreatic ER kinase (PKR)-like ER kinase
PI: propidium iodide
TM: tunicamycin
UPR: unfolded protein respond.
