# Supplementary figures and images for: Araloside C Prevents Hypoxia/Reoxygenation-Induced Endoplasmic Reticulum Stress via Increasing Heat Shock Protein 90 in H9c2 Cardiomyocytes
Source: Front Pharmacol. 2018 Apr 17;9:180. doi: 10.3389/fphar.2018.00180 (PMC5914297; doi:10.3389/fphar.2018.00180)

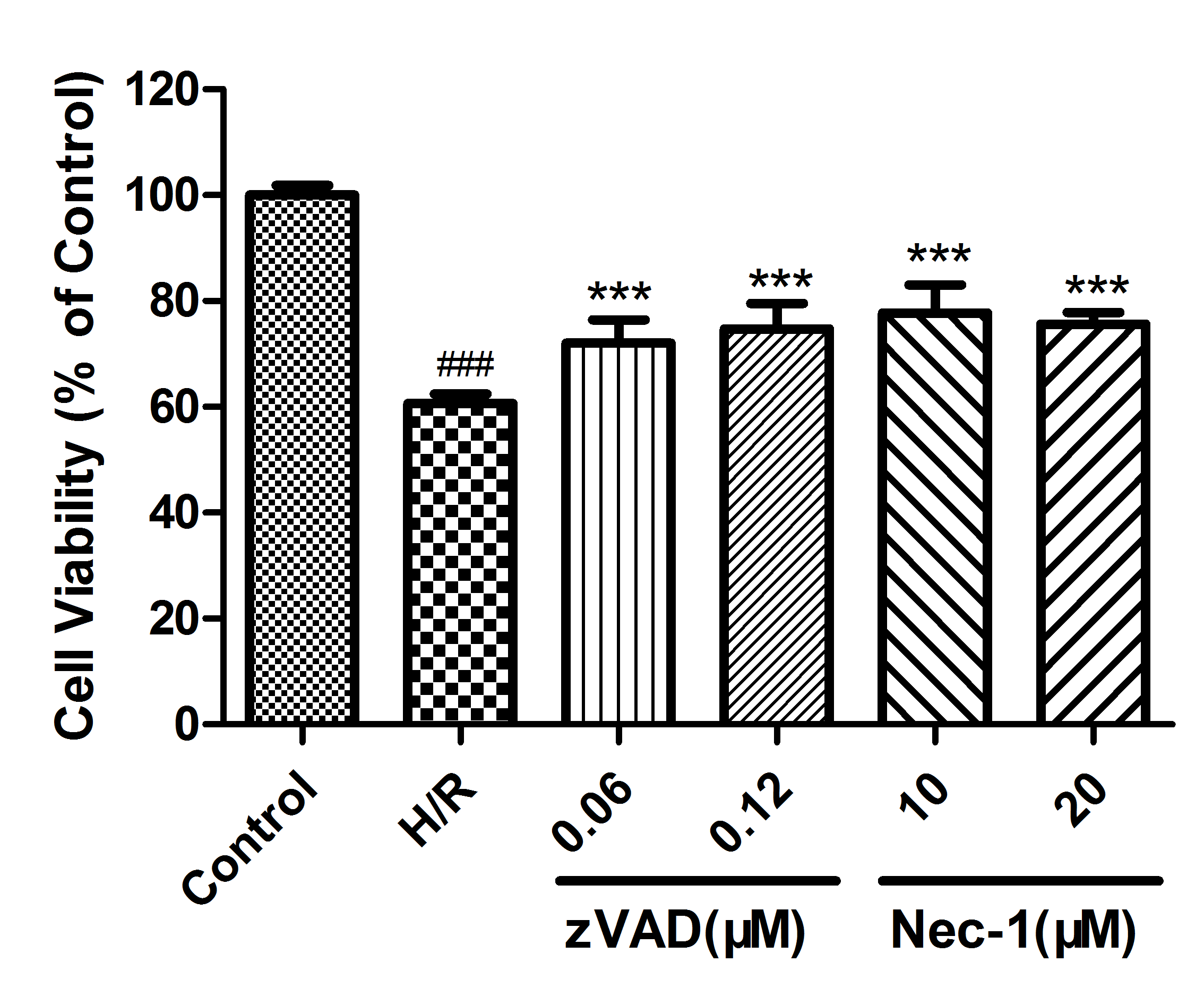

Supplement: FIGURE S1 — Effects of zVAD-fmk (zVAD, apoptosis inhibitor) and Necrostatin-1 (Nec-1, necrosis inhibitor) on H/R-induced cell injury. H9c2 cardiomyocytes were treated with different concentrations of zVAD and Nec-1 for 12 h, followed by 6 h of hypoxia and 12 h of reoxygenation. Cell viability was measured by MTT assays. The values are expressed as the mean ± SD four independent experiments. ###P < 0.001 vs. Control; ∗∗∗P < 0.001 vs. H/R group. [file Image_1.TIF]

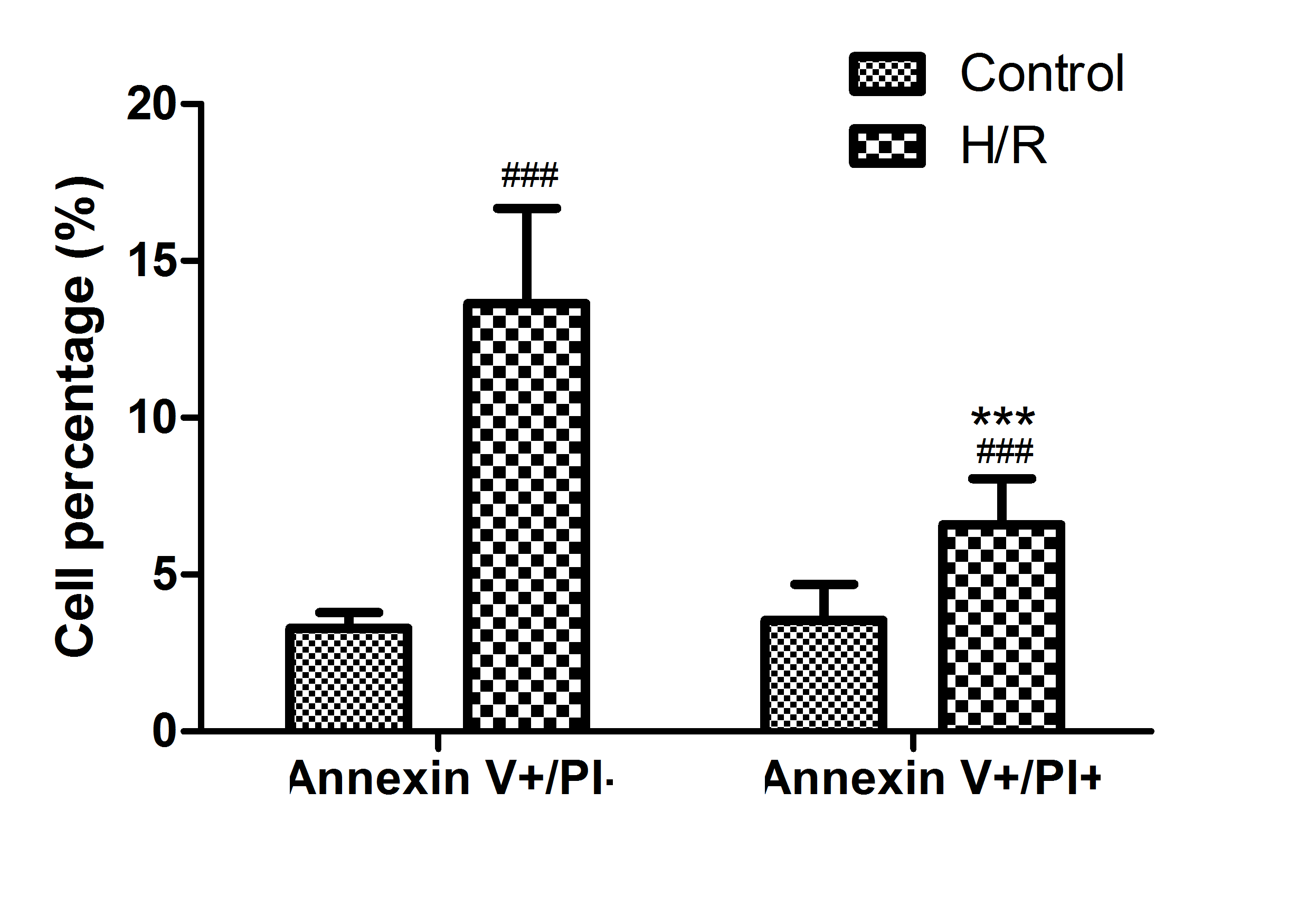

Supplement: FIGURE S2 — Quantitative analysis of the percentages of Annexin V positive and PI negative (AV+/PI-) cells and Annexin V positive and PI positive (AV+/PI+) cells with or without H/R treatment. Annexin V/PI assay kit was used and cellular fluorescence was determined by flow cytometer. The values are expressed as the mean ± SD four independent experiments. ###P < 0.001 vs. Control; ∗∗∗P < 0.001 vs. H/R group in AV+/PI-) cells. [file Image_2.TIF]

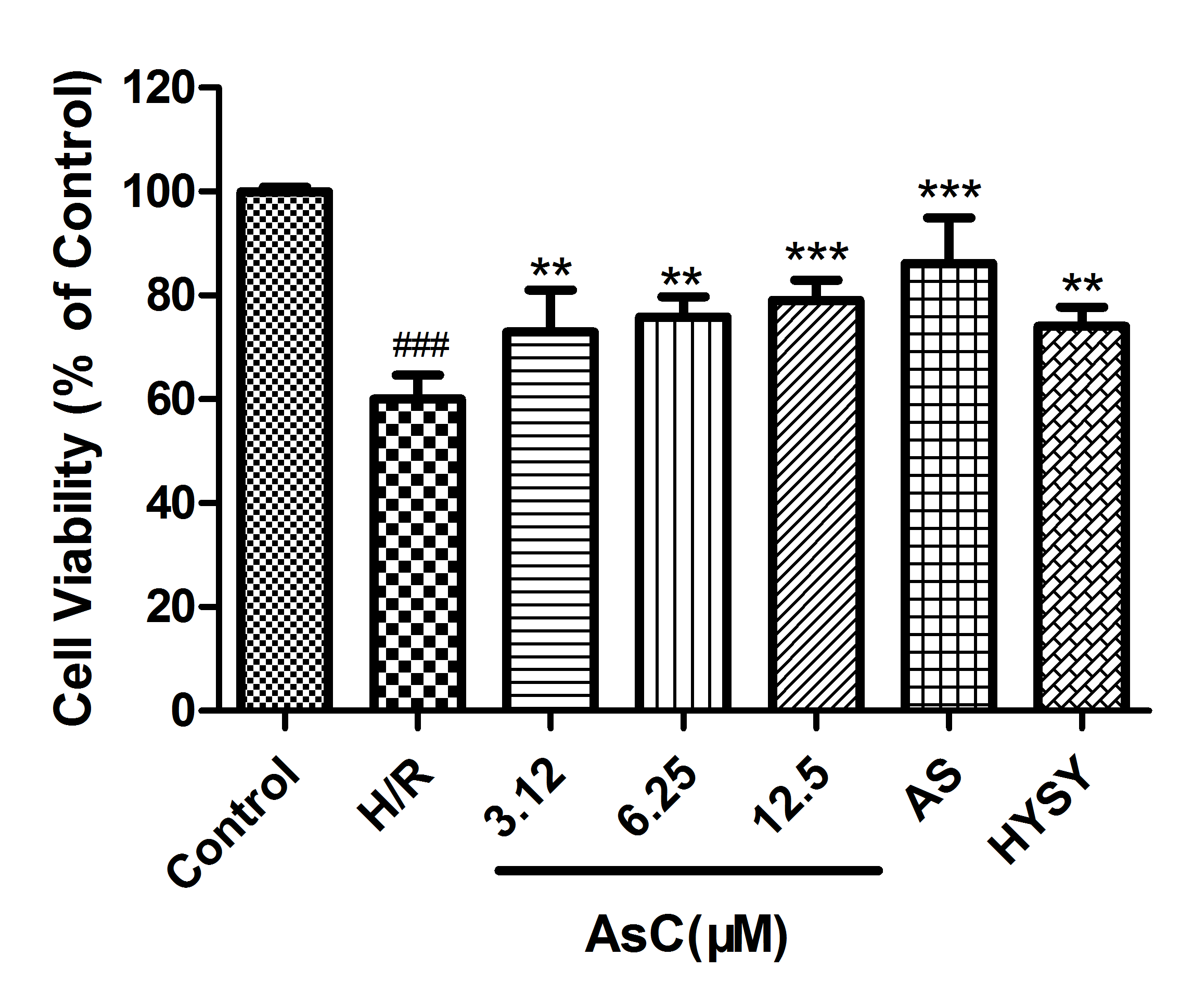

Supplement: FIGURE S3 — Effects of AsC on H/R-induced cell injury. H9c2 cardiomyocytes were treated with different concentrations of AsC and Nec-1 for 12 h, followed by 6 h of hypoxia and 12 h of reoxygenation. Total saponins of Aralia elata (AS, 12.5 μg/ml) and hydroxysafflor yellow A (HSYA, 3 μM) were used as positive controls. Cell viability was measured by MTT assays. The values are expressed as the mean ± SD four independent experiments. ###P < 0.001 vs. Control; ∗∗P < 0.01 vs. H/R group, ∗∗∗P < 0.001 vs. H/R group. [file Image_3.TIF]
